# Human milk lactoferrin variation in relation to maternal inflammation and iron deficiency in northern Kenya

**DOI:** 10.1002/ajhb.23812

**Published:** 2022-10-01

**Authors:** Masako Fujita, Katherine Wander, Nerli Paredes Ruvalcaba, Amelia Ngozi Odo

**Affiliations:** ^1^ Department of Anthropology Michigan State University East Lansing Michigan USA; ^2^ Biomarker Laboratory for Anthropological Research Michigan State University East Lansing Michigan USA; ^3^ Department of Anthropology Binghamton University (SUNY) Binghamton New York USA; ^4^ Laboratory for Anthropometry and Biomarkers Binghamton University Binghamton New York USA; ^5^ Department of Human Kinetics and Health Education University of Nigeria Nsukka Nigeria

## Abstract

**Background:**

Milk lactoferrin is a multi‐functional, iron‐binding glycoprotein with immunomodulatory effects, protecting infants against infectious diseases.

**Aims:**

This study explored how maternal inflammation/infection and iron‐deficiency anemia (IDA) might influence human milk lactoferrin. Lactoferrin might be elevated with maternal inflammation resulting from infectious disease processes. Conversely, lactoferrin might decrease with IDA, corresponding to scarce maternal iron for transfer in milk. In these two hypothesized scenarios, the degree of lactoferrin elevation or decrease might vary with infant vulnerability to infectious diseases or malnutrition. Alternatively, lactoferrin might be unassociated with inflammation/infection or IDA if mothers could buffer it against these conditions.

**Materials & Methods:**

We used cross‐sectional data from Ariaal mothers of northern Kenya (*n* = 200) to evaluate associations between milk lactoferrin and maternal inflammation/infection, IDA, infant age/sex, and the mother‐infant variable interactions in multivariate regression models.

**Results:**

Maternal inflammation was associated with higher lactoferrin for younger infants (<~5 months of age) but with lower lactoferrin for older infants. Maternal IDA was unassociated with lactoferrin alone or in interaction with infant variables.

**Discussion & Conclusion:**

Results suggest that mothers of vulnerable young infants deliver more lactoferrin when they have inflammation/infection but mothers with older infants do not, and that maternal delivery of lactoferrin is unaffected by their IDA. Longitudinal research should verify these findings.

## 1 INTRODUCTION

Milk lactoferrin concentrations vary widely in the existing literature, but the factors underlying this variation are incompletely understood (Villavicencio et al., 2017). This study explored maternal and infant factors associated with variation in milk lactoferrin, an iron‐binding protein with antimicrobial effects that can protect infants against infectious diseases (Kell et al., 2020; Liu & Newburg, 2013).

Some studies report associations between maternal infections or allergies and elevated milk lactoferrin, yet others report lack thereof, as reviewed in (Villavicencio et al., 2017). Furthermore, existing data are too limited to discern the influence of maternal iron nutrition. Being an iron‐binding protein, lactoferrin is capable of nourishing infants with maternal iron (Miller, 2016). Although much of iron transfer occurs during gestation, lactation is an opportunity to bolster infant iron status particularly in mature milk (Hirai et al., 1990; Miller, 2016). Therefore, milk lactoferrin levels might be proportionate to maternal iron availability.

Alternatively, lactoferrin might be buffered against the effect of maternal infection or malnutrition, similarly to other milk bioactives, should natural selection have favored their stability over fluctuation (Fujita et al., 2019; Hinde & Milligan, 2011; Miller et al., 2013; Quinn et al., 2012).

Moreover, the role of infant characteristics such as sex and age, and how these may interact with maternal factors, are underexplored. The existing research reports the importance of lactation stage (correlated with infant age), but the information tends to be limited to the first 1–2 months (Villavicencio et al., 2017) rather than the first 1–2 years, more aligned with lactation duration in places where long‐term breastfeeding is the norm. Research on the effect of infant sex on milk lactoferrin is extremely limited (Liu et al., 2019; Mwila‐Kazimbaya et al., 2017) even though sexes differ in sensitivity to stressors, including infectious disease mortality (Stinson, 1985). The dearth of information on milk lactoferrin variation is particularly noteworthy for the African continent (Villavicencio et al., 2017).

### 1.1 Objectives and hypotheses

We investigated the variation in milk lactoferrin in relation to maternal iron nutrition and inflammation, and how infant characteristics may be involved through the study of associations.

We had three sets of predictions:

1. If mothers buffer milk lactoferrin against their own stressors, milk lactoferrin *would not be* lower among mothers with inflammation or iron deficiency.
2. If lactoferrin delivers maternal iron to infants proportionately to maternal status, milk lactoferrin *would be* lower with maternal iron deficiency. This association would be attenuated for:
  - Younger infants (requiring mitigation against effects of iron shortfalls).
  - Male infants (requiring mitigation against greater sensitivity to stressors from iron shortfalls).
3. If mothers enhance immune protection for infants when undergoing infection, milk lactoferrin would be higher with elevated maternal inflammation (indicative of infectious disease processes). The association would be stronger for:
  - Younger infants (requiring greater protection due to physiological vulnerability from more rapid growth and underdeveloped immune systems).
  - Male infants (requiring greater protection due to greater sensitivity to adverse effects of stressors)

## 2 MATERIALS AND METHODS

We used cross‐sectional data from 200 Ariaal mother‐infant dyads of northern Kenya (available at DOI: 10.5281/zenodo.6808381) to evaluate the associations between milk lactoferrin and maternal inflammation, iron‐deficiency anemia (IDA), and infant age and sex, and the interactions in the mother‐infant dyads. The data originate in the context of a severe drought of 2006 (Fujita, 2008) when mothers varied widely in their iron and immune status (Fujita, 2008; Fujita et al., 2018), suitable for the present study objectives. The institutional review boards of the University of Washington and Kenya Medical Research Institute approved the original data/specimen collection. The lactoferrin determination using de‐identified milk specimens required no further approvals.

Lactoferrin concentrations were estimated by ELISA (Biovendor RD194334200R) of cryogenically archived foremilk specimens (one per participant) collected the morning after an overnight fast, in 2019 in the Biomarker Laboratory for Anthropological Research at Michigan State University. The intra‐ and inter‐assay CVs for kit controls were respectively ≤5% and ≤12% across six plates.

Other variables were available from our previous research (Fujita, 2008; Fujita et al., 2018); elevated C‐reactive protein (CRP >5 mg/L) per (Brindle et al., 2010) defined maternal inflammation, low hemoglobin (<12 g/L per HemoCue Hb201+) co‐occurring with high DBS transferrin receptor (>5 mg/L per Ramco kit TFC‐94) defined maternal IDA, and Micro‐BCA protein assay (Thermo, Cat.23235) characterized milk total protein. Infant age and sex were from interviews.

We constructed multivariate regression models for natural log‐transformed lactoferrin. Predictors included maternal inflammation, maternal IDA, infant sex and age, and the interaction between mother‐infant variables. Infant age tertiles rather than continuous age were used to model the trends across infant age, in light of the number of terms in models for the sample size. Models were adjusted for maternal age and parity and milk total protein.

We used Stata v.15 for statistical computation and *p* < .05 as the cutoff for reporting associations.

## 3 RESULTS

IDA and inflammation were prevalent; nearly one in five mothers had either condition (Table S1). In regression models (Table 1), maternal IDA was unassociated with lactoferrin, alone or in interaction with infant characteristics. The effect of elevated CRP was negligible in the main‐effect model (Model 1). Inclusion of the interaction between infant age and CRP (Model 2) revealed a positive association between CRP and milk lactoferrin in the youngest infant‐age tertile and an inverse association in older tertiles. Infant sex had no association with lactoferrin either on its own or interactively with CRP (Model 3).

**TABLE 1:** Regression models for natural log‐transformed lactoferrin

	Model 1			Model 2			Model 3		
Variables	Coef.	Beta	*p*	Coef.	Beta	*p*	Coef.	Beta	*p*
Mother									
Iron deficiency anemia^a^	−0.058	−0.046	0.414	−0.071	−0.056	0.311	−0.059	−0.047	0.405
CRP >5 mg/L	−0.017	−0.013	0.811	0.263	0.207	0.018	−0.063	−0.050	0.553
Infant									
Age tertile 1 (≤5.3 mo, Ref.)^b^									
Age tertile 2 (5.5–10.7 mo)	−0.008	−0.008	0.907	0.062	0.061	0.371	−0.004	−0.004	0.955
Age tertile 3 (10.7–19.5 mo)	0.086	0.085	0.195	0.160	0.159	0.024	0.089	0.088	0.183
Male	0.035	0.037	0.496	0.016	0.017	0.748	0.022	0.023	0.699
Infant‐Mother Interaction									
Age tertile 2 × CRP >5				−0.473	−0.195	0.008			
Age tertile 3 × CRP >5				−0.436	−0.226	0.006			
Male × CRP >5							0.081	0.051	0.565
Adjustment									
Mother age	−0.002	−0.036	0.668	−0.003	−0.044	0.586	−0.002	−0.033	0.693
Mother parity	−0.015	−0.072	0.385	−0.014	−0.064	0.431	−0.016	−0.074	0.377
ln (Milk total protein)	1.894	0.675	0.000	1.832	0.653	0.000	1.892	0.674	0.000

*Note*: *n* = 200; Model *p* < 0.0001 for all models; Adjusted *R* ^2^ 0.43, 0.45, 0.42; Mean VIF 1.51, 1.86, 1.82.

^a^ There was no interaction between iron deficiency anemia and infant characteristics.

^b^ Results were similar when infant age was modeled as tertiles or continuous variable.

**FIGURE 1:**
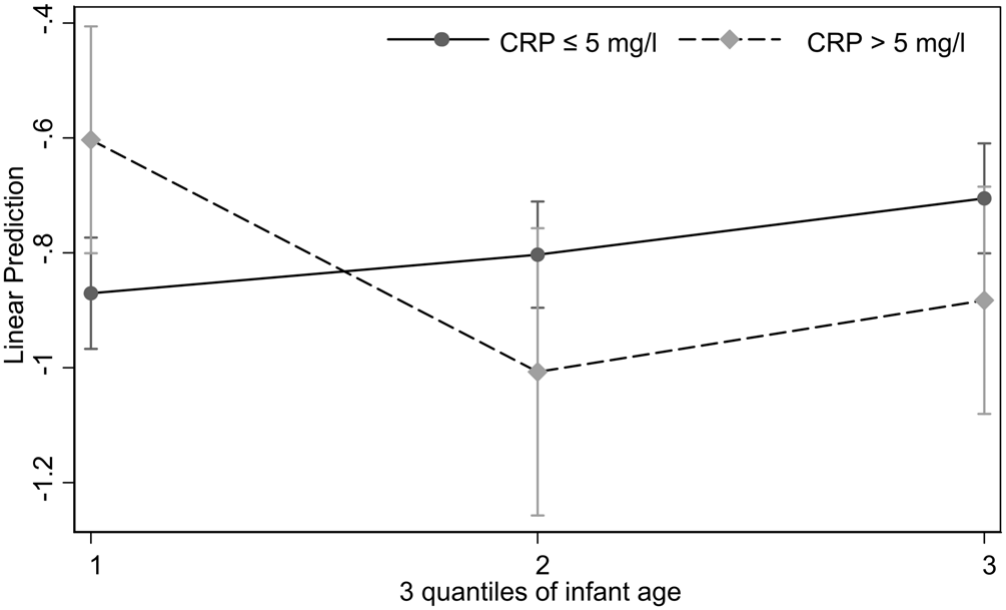
Predictive margin of maternal inflammation (CRP >5 mg/L) on natural log‐transformed lactoferrin across infant age tertiles in Model 2. Among mothers with normal CRP, lactoferrin is lowest for youngest infants and gradually increases as infant age advances. In contrast, among mothers with elevated CRP, lactoferrin is highest for the youngest infants and decreases as infant age advances to the level lower than those among normal CRP mothers. These patterns suggest that maternal inflammation is associated with elevated lactoferrin for youngest infants, here approximately from 1 through 5 months of age (mean ± SD of anti‐log predicted values: 0.70 ± 0.28 vs. 0.50 + 0.12 g/L for 68 mothers), but maternal inflammation is associated with lower lactoferrin for older infants (0.32 ± 0.06 vs. 0.46 ± 0.17 g/L from 6 through 11 months for 65 mothers; 0.34 ± 0.12 vs. 0.48 ± 0.15 g/L from 11 through 19 months of age for 67 mothers.) The overall lactoferrin mean was 0.51 ± 0.26, ranging from 0.12 and 1.54 g/L for 200 mothers.

Figure 1 illustrates the infant age‐CRP interaction (per Model 2). Lactoferrin for youngest tertile infants was higher if mothers had elevated CRP, but this was not the case for older infants. Results were similar when infant age was modeled as a continuous variable.

## 4 DISCUSSION

Our study has limitations, most notably our cross‐sectional data. We modeled the longitudinal change in lactoferrin using the cross‐sectional variable infant age rather than monitoring mother–infant dyads across time, which could have revealed different patterns.

Nevertheless, this study provides insights for milk lactoferrin variation among a sizable group of mothers facing heightened nutritional and infectious disease stress. That milk lactoferrin was unassociated with maternal IDA suggests a buffering effect against iron scarcity, similar to milk lactose and secretory immunoglobulin A among the Ariaal (Fujita et al., 2018; Fujita et al., 2019). This has rarely been investigated in other populations.

In our study, the association between maternal inflammation and elevated lactoferrin became clear only when the model accounted for interaction between maternal inflammation and infant age. It appears then that maternal inflammation, as part of their immune response to some stressors, likely infectious disease stress, has a positive influence on lactoferrin delivery particularly when raising younger infants but not beyond that period. Earlier studies on maternal infection and milk lactoferrin have rarely accounted for the interaction between infant age and maternal infection, and this may have contributed to the conflicting associations or lack thereof in the existing literature (Villavicencio et al., 2017).

That mothers of young infants delivered more lactoferrin when they have inflammation/infection supports our expectation that lactoferrin would be elevated with maternal infection and for vulnerable young infants. However, it falls short of our expectation of elevated lactoferrin for infants of mothers with infection more generally. It is unclear why lactoferrin would decrease for older infants of mothers with elevated inflammation, because they are likely to have heightened risk for infection and so they have elevated need for immune protection. It may be that sustained elevation of lactoferrin is unfeasible due to energetic cost to the mother, or to some hazards to the infant—excessive inflammatory activity (Liu & Newburg, 2013) in the infant gut might adversely affect microflora and nutrient absorption. However, the present study with cross‐sectional data cannot rule out the possibility that the decrease in lactoferrin is an artifact of modeling change across time using cross‐sectional data. It nonetheless points clearly to the need to consider how maternal buffering may play out in complex ways (Fujita et al., 2018, 2019).

## 5 CONCLUSION

The interaction within mother‐infant dyads merits investigation for better understanding sources of variation in milk lactoferrin. Longitudinal research is needed to verify the interactive effect of infant age and maternal inflammation on lactoferrin. As well, future research should clarify the implications of this interaction for infant infectious disease risk.

## CONFLICT OF INTEREST

The authors declare no conflict of interest.

## Supporting information


**TABLE S1** Sample Characteristics (n = 200)

## Data Availability

The data that support the findings of this study are openly available in Zenodo at https://zenodo.org/, reference number 10.5281/zenodo.6808381.

## References

[ajhb23812-bib-0001] Brindle, E. , Fujita, M. , Shofer, J. , & O'Connor, K. A. (2010). Serum, plasma, and dried blood spot high‐sensitivity C‐reactive protein enzyme immunoassay for population research. Journal of Immunological Methods, 362(1–2), 112–120. 10.1016/j.jim.2010.09.014 20850446 PMC2964394

[ajhb23812-bib-0002] Fujita, M. (2008). *An epidemiological and evolutionary investigation of mother‐offspring vitamin A transfer* [Unpublished PhD thesis]. University of Washington. Seattle, WA.

[ajhb23812-bib-0003] Fujita, M. , Paredes Ruvalcaba, N. , Wander, K. , Corbitt, M. , & Brindle, E. (2018). Buffered or impaired: Maternal anemia, inflammation and breast milk macronutrients in northern Kenya. American Journal of Physical Anthropology, 168(2), 329–339. 30575959 10.1002/ajpa.23752 PMC6352968

[ajhb23812-bib-0004] Fujita, M. , Wander, K. , Paredes Ruvalcaba, N. , & Brindle, E. (2019). Human milk sIgA antibody in relation to maternal nutrition and infant vulnerability in northern Kenya [article]. Evolution Medicine and Public Health, 2019(1), 201–211. 10.1093/emph/eoz030 32405414 PMC7216193

[ajhb23812-bib-0005] Hinde, K. , & Milligan, L. A. (2011). Primate milk: Proximate mechanisms and ultimate perspectives. Evolutionary Anthropology, 20(1), 9–23. 10.1002/evan.20289 22034080

[ajhb23812-bib-0006] Hirai, Y. , Kawakata, N. , Satoh, K. , Ikeda, Y. , Hisayasu, S. , Orimo, H. , & Yoshino, Y. (1990). Concentrations of lactoferrin and iron in human milk at different stages of lactation. Journal of Nutritional Science and Vitaminology, 36(6), 531–544. 2097325 10.3177/jnsv.36.531

[ajhb23812-bib-0007] Kell, D. B. , Heyden, E. L. , & Pretorius, E. (2020). The biology of lactoferrin, an iron‐binding protein that can help defend against viruses and bacteria. Frontiers in Immunology, 11, 1221. 10.3389/fimmu.2020.01221 32574271 PMC7271924

[ajhb23812-bib-0008] Liu, B. , Gu, F. J. , Ye, W. H. , Ren, Y. P. , & Guo, S. T. (2019). Colostral and mature breast milk protein compositional determinants in Qingdao, Wuhan and Hohhot: Maternal food culture, vaginal delivery and neonatal gender. Asia Pacific Journal of Clinical Nutrition, 28(4), 800–811. 10.6133/apjcn.201912_28(4).0017 31826378

[ajhb23812-bib-0009] Liu, B. , & Newburg, D. S. (2013). Human milk glycoproteins protect infants against human pathogens. Breastfeeding Medicine, 8(4), 354–362. 10.1089/bfm.2013.0016 23697737 PMC3725943

[ajhb23812-bib-0010] Miller, E. M. (2016). The reproductive ecology of iron in women. American Journal of Physical Anthropology, 159, 172–195. 10.1002/ajpa.22907 26808104

[ajhb23812-bib-0011] Miller, E. M. , Aiello, M. O. , Fujita, M. , Hinde, K. , Milligan, L. , & Quinn, E. A. (2013). Field and laboratory methods in human milk research [Research Support, U.S. Gov't, Non‐P.H.S. Review]. American Journal of Human Biology, 25(1), 1–11. 10.1002/ajhb.22334 23109280

[ajhb23812-bib-0012] Mwila‐Kazimbaya, K. , Garcia, M. P. , Bosomprah, S. , Laban, N. M. , Chisenga, C. C. , Permar, S. R. , Simuyandi, M. , Munsaka, S. , & Chilengi, R. (2017). Effect of innate antiviral glycoproteins in breast milk on seroconversion to rotavirus vaccine (Rotarix) in children in Lusaka, Zambia. PLoS One, 12(12), e0189351. 10.1371/journal.pone.0189351 29284036 PMC5746212

[ajhb23812-bib-0013] Quinn, E. A. , Largado, F. , Power, M. , & Kuzawa, C. W. (2012). Predictors of breast milk macronutrient composition in filipino mothers. American Journal of Human Biology, 24(4), 533–540. 10.1002/ajhb.22266 22434662

[ajhb23812-bib-0014] Stinson, S. (1985). Sex differences in environmental sensitivity during growth and development. American Journal of Physical Anthropology, 28(S6), 123–147.

[ajhb23812-bib-0015] Villavicencio, A. , Rueda, M. S. , Turin, C. G. , & Ochoa, T. J. (2017). Factors affecting lactoferrin concentration in human milk: How much do we know? Biochemistry and Cell Biology, 95(1), 12–21. 10.1139/bcb-2016-0060 28075610 PMC5551053

