# Supplementary material for: Human milk lactoferrin variation in relation to maternal inflammation and iron deficiency in northern Kenya
Source: Am J Hum Biol. 2022 Oct 1;34(12):e23812. doi: 10.1002/ajhb.23812 (PMC10078565; doi:10.1002/ajhb.23812)
Supplement: Supplementary file 1 — TABLE S1 Sample Characteristics (n = 200) [file AJHB-34-0-s001.docx]

**Supplemental Information**

Table S1 Sample Characteristics (n=200)

|  | Mean (SD) or n (%) | min, max |
| --- | --- | --- |
| *Mother* |  |  |
| Age, years | 28 (6.8) | 18, 46 |
| Parity | 3.7 (2.2) | 1, 12 |
| Iron Deficiency Anemia | 35 (18) |  |
| CRP > 5 mg/l | 34 (17) |  |
| *Infant* |  |  |
| Age, months | 8 (4.5) | 0.8, 19.5 |
| Tertile 1 | 68 (34.0) | 0.8, 5.3 |
| Tertile 2 | 65 (32.5) | 5.5, 10.7 |
| Tertile 3 | 67 (33.5) | 10.7, 19.5 |
| Male | 112 (56) |  |
| *Milk* |  |  |
| Total protein, g/dl | 0.98 (0.17) | 0.65, 1.62 |
| Ln (total protein) | -0.03 (0.17) | -0.43, 0.48 |
| Lactoferrin g/l | 0.51 (0.26) | 0.12, 1.54 |
| Ln (lactoferrin) | -0.80 (0.48) | -2.08, 0.43 |
